# Bacterial and Phytoplankton Responses to Nutrient Amendments in a Boreal Lake Differ According to Season and to Taxonomic Resolution

**DOI:** 10.1371/journal.pone.0038552

**Published:** 2012-06-08

**Authors:** Sari Peura, Alexander Eiler, Minna Hiltunen, Hannu Nykänen, Marja Tiirola, Roger I. Jones

**Affiliations:** 1 Department of Biological and Environmental Science, University of Jyväskylä, Jyväskylä, Finland; 2 Department of Ecology and Genetics, Uppsala University, Uppsala, Sweden; 3 Department of Biology, University of Eastern Finland, Joensuu, Finland; Argonne National Laboratory, United States of America

## Abstract

Nutrient limitation and resource competition in bacterial and phytoplankton communities may appear different when considering different levels of taxonomic resolution. Nutrient amendment experiments conducted in a boreal lake on three occasions during one open water season revealed complex responses in overall bacterioplankton and phytoplankton abundance and biovolume. In general, bacteria were dominant in spring, while phytoplankton was clearly the predominant group in autumn. Seasonal differences in the community composition of bacteria and phytoplankton were mainly related to changes in observed taxa, while the differences across nutrient treatments within an experiment were due to changes in relative contributions of certain higher- and lower-level phylogenetic groups. Of the main bacterioplankton phyla, only *Actinobacteria* had a treatment response that was visible even at the phylum level throughout the season. With increasing resolution (from 75 to 99% sequence similarity) major responses to nutrient amendments appeared using 454 pyrosequencing data of 16S rRNA amplicons. This further revealed that OTUs (defined by 97% sequence similarity) annotated to the same highly resolved freshwater groups appeared to occur during different seasons and were showing treatment-dependent differentiation, indicating that OTUs within these groups were not ecologically coherent. Similarly, phytoplankton species from the same genera responded differently to nutrient amendments even though biovolumes of the majority of taxa increased when both nitrogen and phosphorus were added simultaneously. The bacterioplankton and phytoplankton community compositions showed concurrent trajectories that could be seen in synchronous succession patterns over the season. Overall, our data revealed that the response of both communities to nutrient changes was highly dependent on season and that contradictory results may be obtained when using different taxonomic resolutions.

## Introduction

Bacteria and phytoplankton are at the base of lake foodwebs acting as 'energy mobilisers ' and determine whether the system is net autotrophic or net heterotrophic [1]. Production by these microbial communities can be limited by either top-down or bottom-up control [2]. Until recently, bottom-up control was believed to be mainly the limitation by only one element at any given time (Liebig 's Law of the Minimum [3]) and the most frequently limiting elements in freshwater lake ecosystems have usually been considered to be carbon or phosphorus for bacteria and phosphorus or nitrogen for phytoplankton [4]–[8]. However, an alternative view is that the Law of the Minimum is not directly applicable to complex natural communities, such that even though single species may be limited by one nutrient at any given time, communities are often co-limited by multiple elements [9], [10].

Several ratios have been suggested to predict the limiting nutrient from various environments and community components (e.g. [11]–[13]). The most widely used ratio, the Redfield ratio of 106 C:16 N:1 P was first observed to broadly describe the typical composition of phytoplankton biomass in the open ocean [14] and, even though it is now widely applied to almost any environment, it might not be appropriate when ratios are determined from nutrient supply rather than accumulated biomass. Instead, the ratio between dissolved inorganic nitrogen (DIN) and phosphorus (DIP) might be a better predictor of the limiting nutrient based on nutrient supply [15].

The bacterial community composition has been shown to follow a synchronous pattern correlated with that of the phytoplankton community across lakes [16] and seasons [16], [17], which might reflect interactions between these communities. Still there is a wide variation in nutrient requirements between organisms and although functional differentiation within bacterial taxa has been recognized [18]–[20], available techniques have not previously enabled detailed resolution of potentially ecologically coherent groups. Hence conclusions regarding nutrient limitation have been drawn for rather broad and diverse groups; for example, that *Actinobacteria* do not respond to carbon [21] and that *Betaproteobacteria* respond to ammonium but not to nitrate [22]. However, while the total community or even broad phyla might be experiencing co-limitation by N and P, the individual community members or populations could be limited by only one element [10], since taxa within a phylum are not necessarily functionally or metabolically coherent. Therefore even bacteria closely related to each other may differ markedly in their elemental composition and nutrient limitation, making any conclusions drawn for high taxonomic levels (e.g. phylum-level) quite arbitrary.

Here we hypothesized that the perceived limiting nutrient for any given group of organisms is dependent on the applied level of taxonomic resolution as well as season. Further, we examined the simultaneous responses of bacterio- and phytoplankton community composition to nutrient manipulation that, to our knowledge has not been previously addressed. To assess these phenomena, we undertook a detailed analysis of bacterial and phytoplankton communities from three nutrient amendment experiments conducted in microcosms with water from a boreal lake during spring, summer and autumn. Experiments were designed to address N and P limitation and especially to provide high taxonomic resolution of bacterioplankton and phytoplankton community responses to nutrient additions. Bacterial community composition was determined using 454 pyrosequencing of 16S rRNA gene amplicons (from pooled replicates) and length heterogeneity analysis of PCR amplified 16S rRNA genes (LH-PCR; [23]) (with unpooled replicates), while phytoplankton (with replicates) community members were identified by microscopy. We show that the response to nutrient amendments is highly dependent on the taxonomic resolution and that the synchronous responses in phytoplankton and bacterioplankton communities are dependent on season.

## Results

### General responses

The three nutrient addition experiments were conducted in early May, July and September 2009 with water from Lake Alinen Mustajärvi in southern Finland (61°12′N, 25°06′E). This lake has been extensively studied and has previously been described in detail [24], [25]. At the time of the nutrient additions, the dissolved organic carbon (DOC) concentration in the lake was artificially increased by 2 mg C L^−1^ by monthly additions of cane sugar as part of a parallel experiment, which supplied the lake bacterial communities with sufficient readily usable carbon available. Water for each mesocosm experiment was taken from the oxic layer of the lake (epilimnion) within 24 hours of the most recent carbon addition, and the N and P concentrations were manipulated in the experimental treatments.

**Figure 1:**
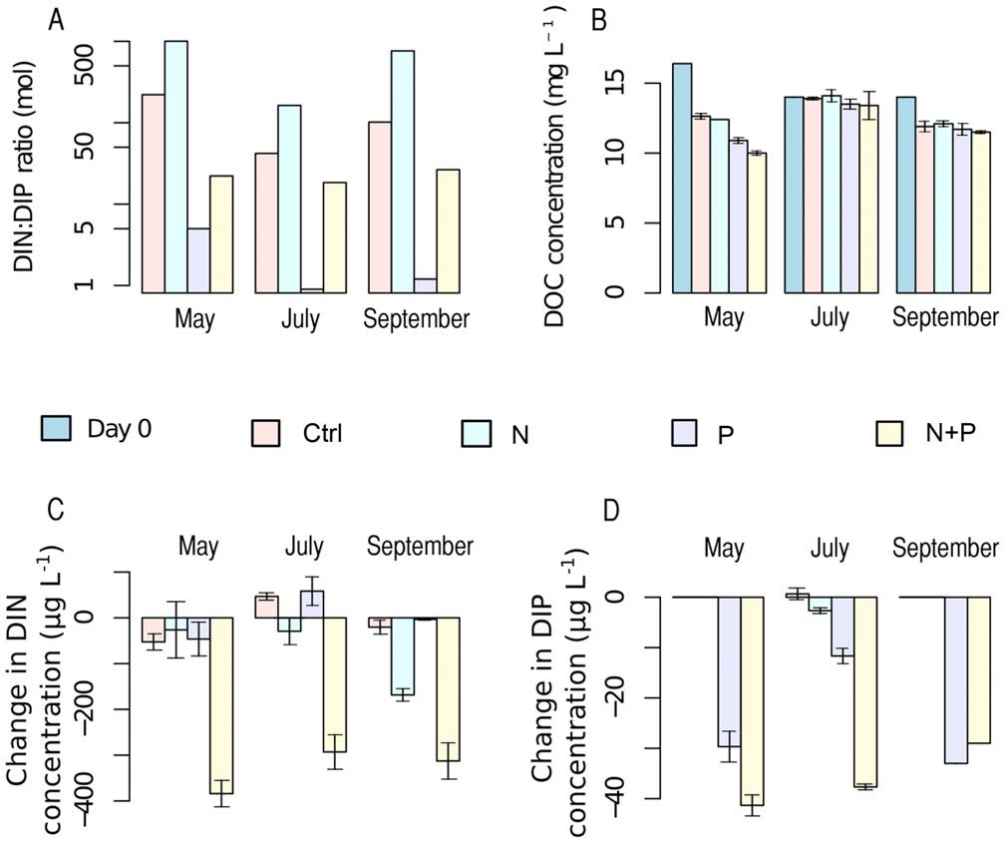
Nutrient and DOC concentrations in the experiments. DIN∶DIP ratios in the lake and in the amended nutrient treatments on day 0 of each experiment, n = 1 for all (A). Change in DOC concentration during experiments, n = 1 for day 0 and n = 4 for day 7 (B). Change in DIN concentration during experiments (C). Change in DIP concentration during experiments (D). In C-D the change is from day 0 to day 7 and n = 4. Error bars represent standard deviation.

The three experiments included four treatments: control (Ctrl), added nitrogen (N), added phosphorus (P) and added nitrogen and phosphorus (N+P), each with three replicates. The DIN∶DIP ratios in the lake and hence also in the Ctrl treatment were 221∶1, 42∶1 and 102∶1 at the beginning of each experiment, respectively (Fig. 1a). According to Ptacnik et al. [15] this would suggest P limitation in the lake at the time of the May and September experiments, but co-limitation by N and P in July. After the nutrient additions, the DIN∶DIP ratio pointed to P limitation in the N treatment and vice versa in the P treatment in all three experiments performed at different seasons, while in the N+P treatment the ratio in May and September was in the range of either co-limitation or no limitation and in the July experiment it was most probably N limited.

**Figure 2:**
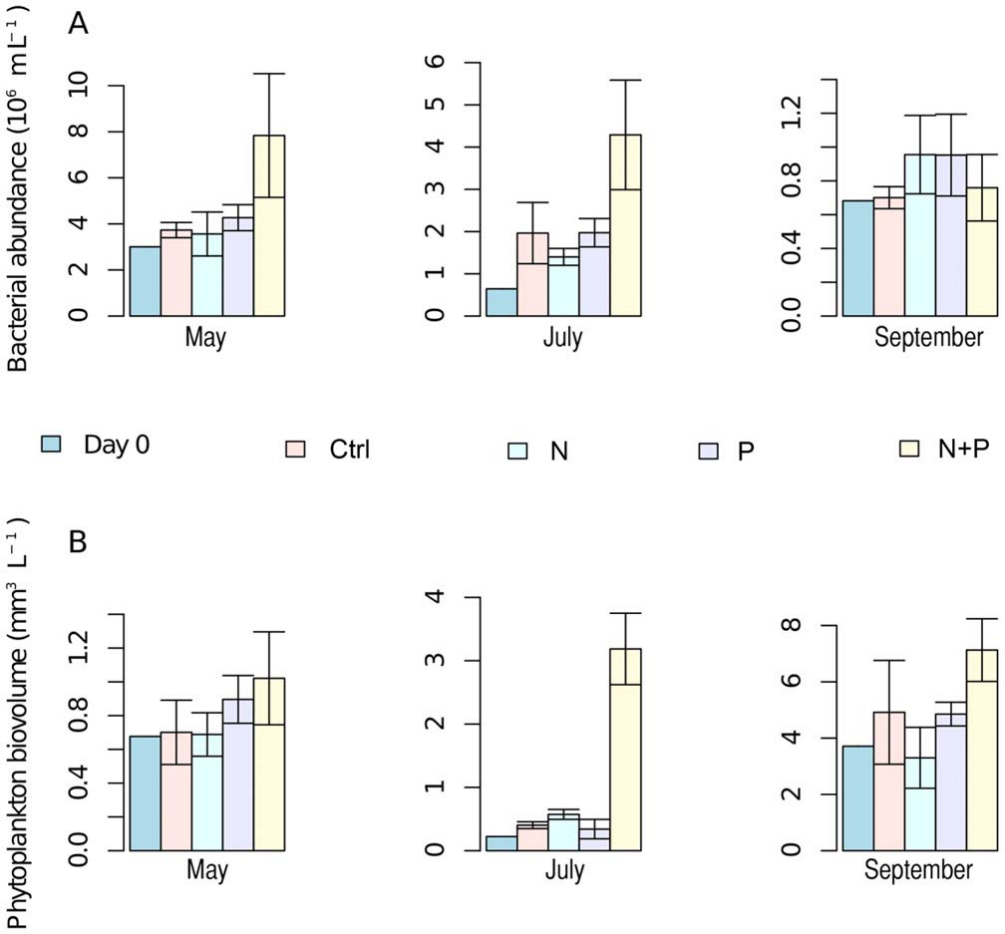
Bacterial abundance (A) and phytoplankton biovolume (B) at the start and at the end of the experiments. Note different Y-axis scales between the panels. In all panels n = 1 for day 0 and n = 4 for day 7. Error bars represent standard deviation.

Changes in DOC, DIN and DIP concentrations were estimated from concentrations at the start and the end of each experiment (Figures 1b–c). A significant decrease in DOC concentration of 4–6 mg C L^−1^ was observed only in the May experiment (χ^2^ = 10.53, p<0.05). DIN concentration decreased in May and September (χ^2^ = 9.36 and 9.70, respectively, p<0.05 for both), mainly in the N+P treatment. DIP concentration decreased in all experiments (χ^2^ = 10.65, 10.49 and 11.0, respectively for May, July and September, p<0.05 for all) in P and N+P treatments. Overall the bacterial abundance in the lake declined towards autumn whereas phytoplankton biovolume increased (Figures 2 a–b), but differences in abundance and biovolume between treatments were minor except for the N+P treatment. Thus, changes in bacterial abundance and phytoplankton biovolume, as well as changes in nutrient concentrations during experiments, suggested co-limitation by N and P for bacteria in May and July and for phytoplankton in July and September. For bacteria in September and for phytoplankton in May some other factor seemed to be limiting growth. Still, overall the community responses, such as changes in biovolumes and abundances, to treatments were generally smaller than the seasonal changes between experiments.

The comparison between 454 pyrosequencing results (based on 97% OTUs) and LH-PCR predictions (based on fragment length and predictions based on the 16S rRNA gene clone library data from Alinen Mustajärvi from 303 clones) showed high similarity between methods in the abundance of major phyla as well as in Morisita-Horn distance matrices (Table 1; procrustes test: R = 0.923, p<0.001). Morisita-Horn metrics were chosen for this study based on its robustness with samples of differing sample size [26], [27] as the acquisition of phytoplankton and LH-PCR data did not allow for resampling. Since the 454 pyrosequencing and LH-PCR gave similar results, LH-PCR replicates were used to examine statistical differences among communities, as 454 pyrosequencing was done from pooled replicates.

**Table 1:** Results from generalized linear models (GLM) relating the proportions of different phyla detected in the 454-pyrosequencing and LH-PCR datasets.

Phyla	df	Slope	R^2^	F	p
*Actinobacteria*	21	1.24	0.78	77.06	<0.001
*Alphaproteobacteria*	21	1.16	0.69	48.98	<0.001
*Betaproteobacteria*	21	0.96	0.65	42.52	<0.001

Slopes, R^2^ indicating the regression coefficient, F statistics and the significance level p are shown.

**Figure 3:**
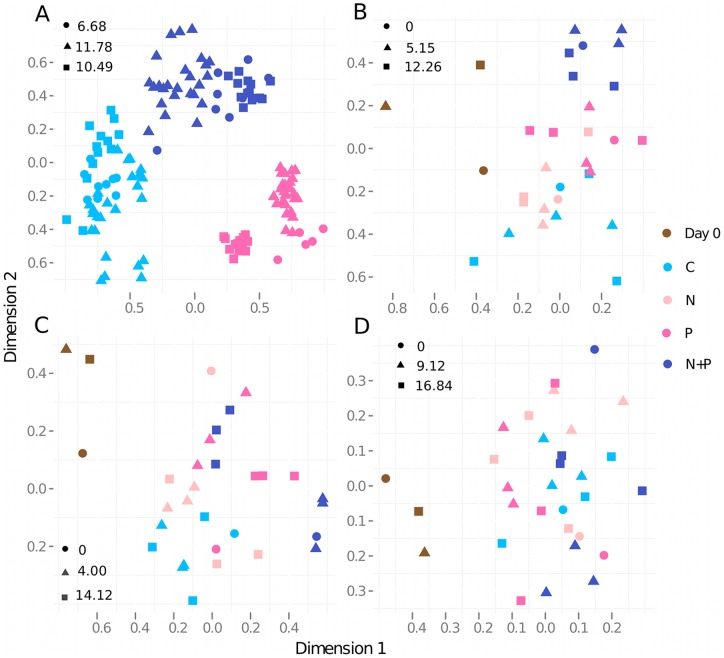
Overlaid non-metric multidimensional scaling plots from 454 pyrosequencing, LH-PCR and phytoplankton data. All three datasets and experiments are overlaid in a single plot with different colours representing experiments (May: light blue, July: dark blue and September: pink) and different shapes representing datasets (A). In panels visualizing May (B), July (C) and September (D) experiments colours represent treatments as in legend and shapes represent datasets. Dissimilarities in community composition were estimated using Morisita-Horn distance metric. Stress values for each community (indicated with shapes) are specified in plots. In all plots • = 454 pyrosequencing, ▴  =  LH-PCR and ▪  =  phytoplankton.

General trends in phytoplankton (identified to species or in some cases genus level) and bacterioplankton community composition were visualized in a single plot (Figure 3a), where NMDS plots derived from three separate dissimilarity matrices (phytoplankton, LH-PCR and 454 pyrosequencing) were overlayed. This is clearly showing a synchronous seasonal succession of both communities, which was also verified by the procrustes test showing high correlation between the datasets (R = 0.750 for 454 vs. phytoplankton and R = 0.632 for LH-PCR vs. phytoplankton, p<0.001 for both). Further, the treatment responses in composition for each experiment were visualized in similar overlays of NMDS plots (Figure 3 b–d), which suggested synchrony in responses to treatments between bacterio- and phytoplankton communities within each experiment. This was also verified with procrustes test, where R was 1.0 for all comparisons (range 0.98–1.0; p<0.01 except for LH-PCR vs. phytoplankton in May and for 454 vs. phytoplankton and LH-PCR in September p<0.05). The dispersion of the bacterial and phytoplankton communities was tested using permutational analysis of multivariate dispersions (also called MJ Anderson's permutated analysis of betadispersion), which was applied on Morisita-Horn based dissimilarity matrices (454 data) [28]. This analysis revealed that the bacterial communities in the September experiment were less dispersed than those in May or July (Table 2), and that the phytoplankton communities in the May experiment were more variable than those in July and September.

**Table 2:** Impact of treatment and experiment to betadispersion of bacterial and phytoplankton communities (upper two lines), and pairwise comparisons of betadispersion of experiments (lower three lines).

	Bacteria		phyto	
Factors	F	p-value	F	p-value
Treatment	1.125	Ns.	0.336	Ns.
Experiment	12.23	<0.001	15.84	<0.001
May-July		Ns.		<0.05
May-September		<0.001		<0.05
July-September		<0.001		<0.001

### Bacterial community responses to treatments

**Figure 4:**
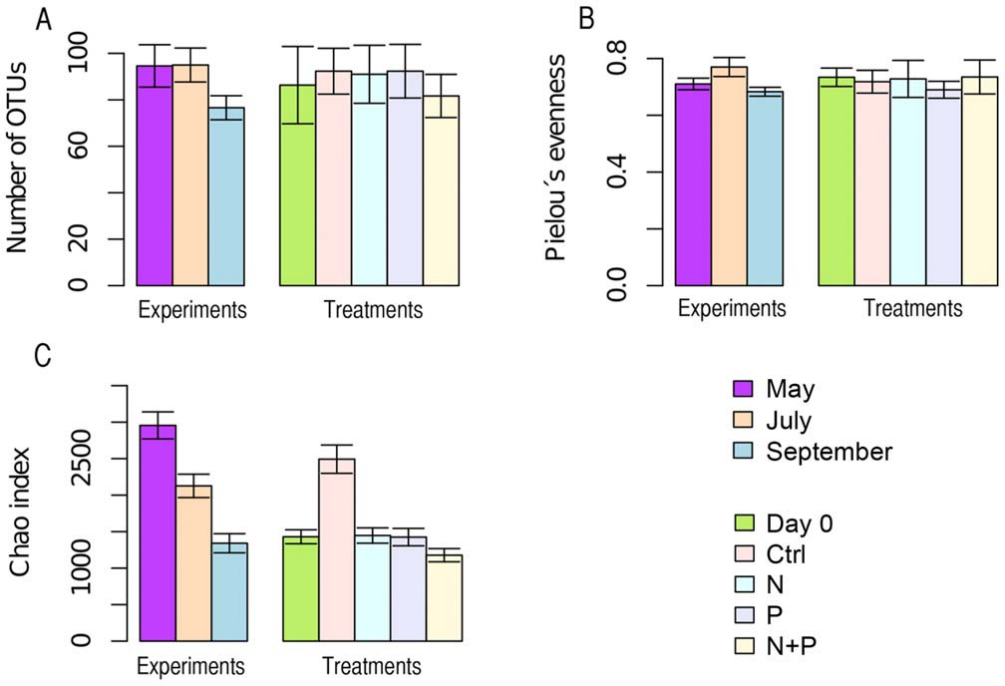
The mean number of OTUs (A), Pielou's evenness index (B) and Chao richness estimate (C) in experiments and treatments. Error bars in (A) and (B) represent standard deviation and in (C) standard error.

454 pyrosequencing of 23 samples of pooled triplicate treatments yielded 60,659 high quality reads from amplicons of the entire V4 region of the 16S rRNA gene. These were assigned to 1622 OTUs by UCLUST [29] with a 97% sequence similarity cutoff loosely corresponding to a bacterial species. The average number of OTUs was 164 per sample (range 133–304) with average OTU numbers for May, July and September experiments being 151, 193 and 149, respectively. According to OTU numbers, diversity in September experiment was lower than in May or July (χ^2^ = 9.48, p<0.01) but there were no differences between treatments (Figure 4a; treatment effect tested across experiments due to missing replication). Furthermore, the Pieloús evenness index was suggesting higher evenness in the community during the July experiment than in May or September (χ^2^ = 11.06, p<0.005) and the Chao richness estimate was higher in July than in May or September, but neither showed differences between nutrient treatments (Figure 4b-c). Additionally, the Morisita-Horn distance between all pair-wise comparisons of treatments and experiments increased linearly with increasing sequencing similarities used for OTU clustering (range 75 to 99% similarity). In other words, the differences between communities increased with increasing resolution (Figure S1; adjusted R^2^ = 0.565 and p<0.001.). At low sequence similarity OTUs could not be assigned to the most-resolved freshwater taxonomic groups (so called tribes; see [30] for definition) since sequence clusters stretched over several taxonomic groups, while with highly resolved sequence clusters with sequence similarity cutoffs >97% splitting of tribes could be observed.

**Figure 5:**
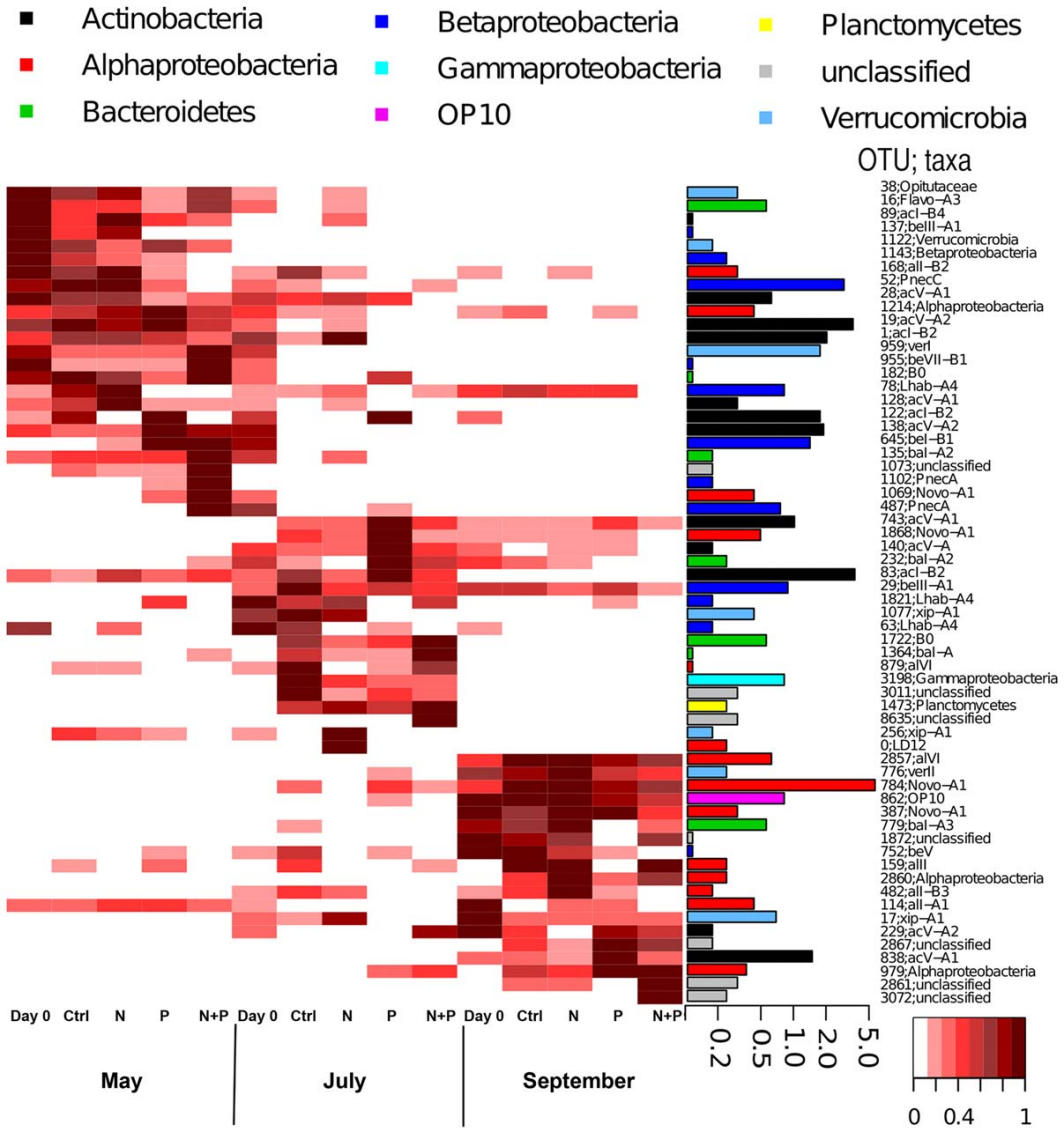
Heatmap visualizing the frequencies of OTUs with a barplot showing their proportions in the entire dataset. Frequencies are given by relativizing OTUs against their maximum read number. The barplots show the actual abundance (% of all reads) of each OTU with logarithmic scale. Taxonomic affiliation of each OTU is given after the identification number.

Using 97% sequence similarity cutoff, clear differences in bacterioplankton community composition could be observed between treatments and seasons (PERMANOVA, Table 3). In a heatmap using resampled values from the pyrosequencing data relativized by the maximum value of each OTU (Figure 5), it can be clearly observed that the differences among seasons were due to OTUs unique to each season. These OTUs specific to seasons responded differently depending on the treatment, resulting in the significant differences in community composition among treatments.

**Table 3:** Results from a permutational multivariate analysis of variance comparing the bacterial (LH-PCR) and phytoplankton communities among seasons (experiments) and after nutrient additions (treatments).

LH-PCR	df	SS	MS	pseudo-F	p
*Season*	2	5.75	2.88	235.88	<0.001
*Treatment*	3	0.54	0.18	14.79	<0.001
*Treatment (season)*	6	0.26	0.04	3.59	<0.001
May (Treatment)	3	0.39	0.13	11.63	<0.001
July (Treatment)	3	0.38	0.13	6.06	<0.005
September (Treatment)	3	0.03	0.01	2.53	Ns.

Phytoplankton	df	SS	MS	pseudo-F	p
*Season*	2	6.99	3.50	948.52	<0.001
*Treatment*	3	0.05	0.02	4.37	<0.05
*Treatment (season)*	6	0.11	0.02	5.10	<0.005
May (Treatment)	3	0.56	0.12	4.06	<0.01
July (Treatment)	3	0.80	0.27	29.75	<0.001
September (Treatment)	3	0.26	0.09	11.81	<0.001

Dissimilarities in community composition were estimated using Morisita-Horn distance metrics. The statistical significance was determined by Monte Carlo simulations (p-value from 10,000 permutations) and *F*-values.

### Bacterioplankton population responses to treatments

OTUs were annotated against a freshwater bacterial sequence database established by Newton et al. [30] and RDP. The heatmap visualizing OTU abundances and taxonomy (Figure 5) showed marked differences between seasons, with the main phyla in the May experiment being *Betaproteobacteria* and *Actinobacteria* whereas by September the community was dominated by *Alphaproteobacteria* (Figure S2). According to LH-PCR, on the phylum level only *Actinobacteria* was showing a clear treatment response (Table 4) with a decrease in N+P treatment in every experiment. At the 97% sequence similarity level, OTUs annotated to the same phylum (*Actinobacteria* and *Alpha*- and *Betaproteobacteria*) did not have uniform treatment responses; instead rather different OTUs belonging to the same phylum increased in abundance in different treatments. For example, in the May experiment actinobacterial OTU128 increased in abundance in the N treatment, while OTU122, also affiliated with *Actinobacteria*, responded to the P treatment. Only a few OTUs showed a treatment response in all experiments (for example, OTUs 78 and 1821, both affiliated with tribe Lhab-A4), but several OTUs did respond in two experiments. An example of this is actinobacterial OTU122 that increased in abundance in the P treatment in May as well as in the July experiment. In the September experiment the treatment responses were overall milder than in May or July as according to LH-PCR there were no differences between treatments (Table 3) and of the phyla the proportion of *Betaproteobacteria,* a major contributor in May, was minor in September and none of the betaproteobacterial OTUs seemed to benefit from the nutrient amendments.

**Table 4:** Results from Kruskal-Wallis tests for experiment (seasonal) and treatment (nutrient addition) effects on the phylum distribution of *Actinobacteria* and *Alpha-* and *Betaproteobacteria*.

Phyla	df	χ^2^	p
*Experiment*			
*Actinobacteria*	2	31.91	<0.001
*Alphaproteobacteria*	2	58.95	<0.001
*Betaproteobacteria*	2	59.06	<0.001
*Treatment*			
*Actinobacteria*	3	26.50	<0.001
*Alphaproteobacteria*	3	0.73	ns
*Betaproteobacteria*	3	5.33	ns

Across seasons 40–60% of the community members belonged to tribes that have been described as typical for freshwater (see [30]) and while this proportion remained relatively constant in most treatments, in the N+P treatment it diminished particularly in the July experiment. OTUs annotated to the same tribe but present during different seasons could show similar responses, as already mentioned for actinobacterial OTU122 belonging to tribe acI-B2. However, there were no uniform treatment responses within season-specific OTUs, that is OTUs belonging to same tribe and found from the same experiment (see, for example, acV-A2 OTUs 19 and 138). Instead OTUs annotated to the same tribe showed varying preferential seasonal occurrence with highly contrasting treatment responses. For example, OTUs annotated to tribes affiliated with *Polynucleobacter* (PnecA and PnecC) were not found at all in the September experiment and further, OTU52 (PnecC) was found to gain exclusively from N+P amendment in July, while in May it was present in all other treatments except in N+P. An example of differentiation within tribes are the two OTUs affiliated with acI-B2 tribe in the May experiment (OTUs 1 and 122), of which one was most abundant in Ctrl and N treatments (OTU1), while the other preferred P treatment (OTU122). Also different OTUs annotated to a single tribe could appear in any experiments from May, July or September; for example, OTU387 annotated to tribe Novo-A1 was found exclusively from the September experiment while OTU784 also belonging to Novo-A1 was present in July and in September.

### Responses of phytoplankton and heterotrophic protists to treatments

**Figure 6:**
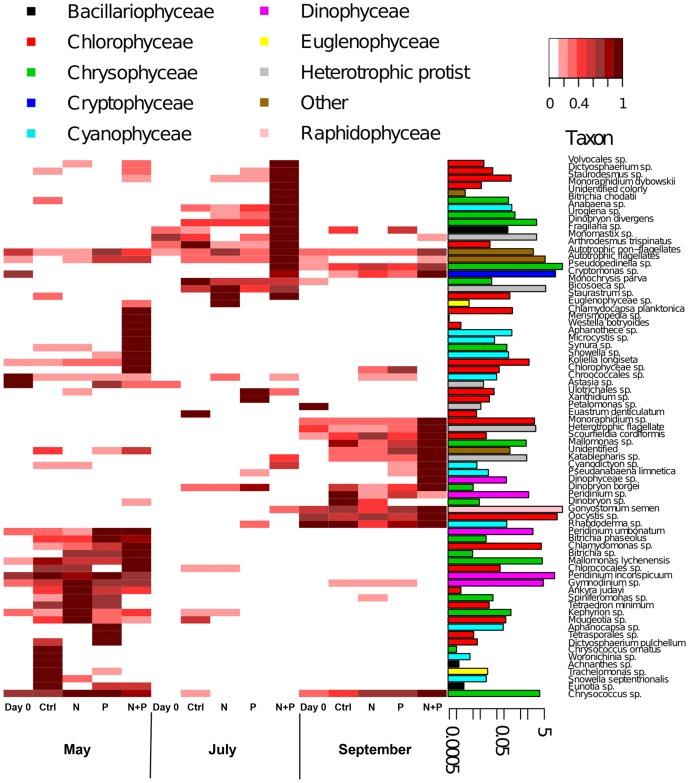
Heatmap visualizing patterns in biovolumes of phytoplankton taxa with the barplots showing their relative contributions to the entire phytoplankton biovolume. Biovolumes were standardized by relativizing each taxon against its maximum biovolume. The barplots show the actual biovolume (% of taxa) of each taxa in logarithmic scale. *  =  *G. semen* contributed 64 % of the whole phytoplankton biovolume in all experiments. To visualize the biovolumes of other taxa, this bar was truncated to same height with the second most abundant taxa, *Pseudopedinella*.

The phytoplankton community composition was dependent on season as well as on treatment in every experiment according to PERMANOVA (Table 3). A heatmap visualizing the changes in phytoplankton (relativized by the maximum value of each taxa; Figure 6) suggested that, similar to bacteria, the differences among seasons were due to taxa unique to each season. In general, Dinophyceae together with Chrysophyceae dominated the phytoplankton in the spring and Raphidophyceae, again together with Chrysophyceae, in summer, while during the autumn experiment there was a bloom of *Gonyostomum semen* (Raphidophyceae). The proportion of potentially mixotrophic taxa was over 50% in all experiments, and their biovolume increased towards autumn (Figure S3; χ^2^ = 23.90, p<0.001), being higher in September than in May or July (p<0.001 for both). The mixotrophic phytoplankton did not seem to benefit from the basic experimental conditions, as their biomass did not increase in Ctrl treatment. In the July and September experiments the biovolumes of mixotrophs responded to treatments (χ^2^ = 8.44 and χ^2^ = 8.13, respectively, p<0.05 for both) being higher in the N+P treatment in July and lower in the N treatment in September.

Community response to treatments visualized in the heatmap was highly dependent on the season and taxa from specific classes did not respond uniformly to the nutrient additions. For example, Dinophyceae-taxa responded only in the May and September experiments, as did most Cyanophyceae-taxa, whereas Chlorophyceae- and Chrysophyceae-taxa grew in all experiments. Overall the response to treatments was most obvious in the N+P treatment, especially in the September experiment. Like the bacterial community, species belonging to the same phylum had differing treatment responses. For example, in the July experiment the biovolume of *Dinobryon divergens* increased in the N+P treatment while the biovolume of *Dinobryon borgei* increased in the P treatment.

Most heterotrophic protists increased following N+P amendment (for example, *Katablepharis* sp. and unidentified heterotrophic flagellates), with few exceptions from other treatments, (for example, *Bicosoeca*-species increased in the N amendment). Overall the biovolume of heterotrophic protists was highest in the July experiment (χ^2^ = 30.58, p<0.001), and in the May and July experiments it was affected by treatments (χ^2^ = 7.82 and χ^2^ = 9.67, respectively, p<0.05 for both) being lower in the Ctrl treatment in May and higher in the N+P treatment in July.

## Discussion

In this study, the bacterioplankton and phytoplankton community compositions in a boreal lake showed concurrent trajectories as synchronous succession patterns over the season and synchronous responses to nutrient additions were observed. This is both visualized in the NMDS plots and statistically verified by procrustes test. Such linked patterns of bacterial and phytoplankton community dynamics have been suggested to be driven directly by phytoplankton-bacteria interactions [16], [31]. These interactions can be mediated through phytoplankton exudates, which are readily available substrates for bacterioplankton growth, but phytoplankton may also shape bacterial communities by the release of antimicrobial agents [32]. Some phytoplankton taxa are capable of mixotrophic growth by selective feeding on bacteria and thus affecting bacterial community composition [33]. Conversely, bacteria may affect the composition of the phytoplankton community as the growth of algae may be affected by certain members of the bacterial community [34]. Although the synchronous trajectories observed here over the season are likely depending on bacteria-phytoplankton interactions, the changes during experiments could also be due to responses to the actual nutrient amendments.

The diversity of bacterial communities was not affected by the treatments, but during the September experiment the diversity was lower than in the other two experiments. This is in contrast with earlier observations where the diversity of bacterial community was lower in spring than in summer or autumn [35]. Furthermore, the strong seasonality may have masked treatment effects on diversity as this was tested across experiments. Also, the seasonal characteristics as well as responses of the communities to nutrient additions were highly dependent on the level of taxonomic resolution. At low taxonomic resolution (bacteria vs. algae), the impact of season on responses to nutrient amendments was contrasting for bacteria and phytoplankton and the initial abundances and biovolumes of the bacterio- and phytoplankton seem to have affected the outcome of the experiments. Bacterial growth was most prominent in the May experiment when the initial bacterial numbers were highest, whereas the highest phytoplankton biovolumes were observed in September, when the growth response of phytoplankton was also most pronounced. Still it should be noted that the response of mixotrophic phytoplankton was also greatest in September, which could have partly concealed the bacterial growth response in autumn.

The changes in community composition of both plankton communities following nutrient amendments were dependent on season, as has been previously reported for lake bacterioplankton [36] and for marine phytoplankton [37]. The bacterial communities in Alinen Mustajärvi appeared to be limited by N and P during spring and summer, but during autumn some other factor was limiting growth. Earlier studies have suggested various scenarios for freshwater bacterioplankton nutrient limitation across seasons, ranging from P limitation during most of or the whole ice free season [5], [38] to spring and autumn N limitation combined with co-limitation by C, N and P during summer [36]. In the September experiment the limiting factor may have been temperature as suggested previously by Vrede [7]. Consistent with our results, freshwater phytoplankton has been suggested to be co-limited by N and P during summer [8], [39], [40], though there are also reports of pure N limitation [5]. In spring the phytoplankton growth appeared to be limited by some other factor than N or P, as was also suggested by Vanni and Temte [39], though also reports of phytoplankton P limitation in spring do exist [8].

Variations in nutrient requirements between organisms was already suggested at the phylum level of bacteria since *Betaproteobacteria* did not seem to benefit at all from nutrient amendments relative to the control in the September experiment whereas abundance of *Alphaproteobacteria* appeared to increase. The overall decline in N concentrations with season (Figure S4) might be one reason, as the *Betaproteobacteria* are frequent in environments with high N concentration, like wastewater treatment plants (e.g. [41]) and the plant rhizosphere [42]. Another reason may be selective grazing by protists. Previous work has shown that increased grazing pressure by heterotrophic flagellates may increase the proportion of filamentous bacteria [17] and thus cause changes in community composition. As many members of *Alphaproteobacteria* have a tendency to form filaments [30] and *Alphaproteobacteria* did increase considerably over the season, it is likely that this increase was at least partly due to ability to resist increasing flagellate grazing. Furthermore, and considering the increases in flagellate abundance especially in the N+P treatment, this could have affected the changes in bacterial community composition during experiments. Since the larger zooplankton were removed from the mesocosms, the increased flagellate growth might have been due to decreased grazing rather than nutrient amendments. However, the flagellate abundance did not increase in the control treatment, which suggests that it was indeed the nutrient amendments that enhanced their growth. Nevertheless, none of the alphaproteobacterial taxa benefitted exclusively from N+P treatment. Conversely, the betaproteobacterial clade betI-A has been observed to be the preferred food source for flagellates [43]–[45], which may also have resulted in the seemingly weak response of certain betaproteobacterial groups. Members of the other phyla with low reactivity in September, the *Actinobacteria,* are typically associated with environments with lower nutrient concentrations [46]. Further, they are considered to be an unattractive food source for grazers [42], [45] and have been found to be less affected by grazing than other bacterial groups [2]. Thus, the increase in flagellate abundance as well as low nutrient concentration in the autumn should have favoured *Actinobacteria.* However, *Alphaproteobacteria* that show similar overall growth characteristics were increasing substantially during the September experiment, including OTUs belonging to *Novosphingobium* (here represented by tribe Novo-A1). Members of this taxon have previously been found to be typical for lakes with high concentration of humic matter [17], [47] and are known for the ability to degrade recalcitrant compounds such as phenols [48]. The more coherent composition of both plankton communities in the September experiment with only the most persistent species proliferating might have resulted from top-down control by increased grazing pressure as indicated by the high numbers of heterotrophic flagellates.

Resolving sequences to tribes was not sufficient to obtain groups responding coherently to nutrient additions as highly divergent patterns were observed among OTUs annotated to the same tribe (see for example OTUs annotated to Novo-A1, PnecA and acV-A1). This highlights the occurrence of ecological differentiation within tribes and further suggests differentiation into divergent functional and also temporal groups with dissimilar resource requirements. This trend was further emphasized when treatment-induced differences increased with increasing OTU-resolution. Even up to the 99% resolution level there was still no indication of a deviation from a linear increase in community distances among pairwise comparisons of treatments indicating insufficient resolution provided by 16SrRNA amplicons. This has already been suggested by niche partitioning among strains of *Polynucleobacter necessarius asymbioticus* in respect to pH, conductivity, DOC and oxygen concentration [20] and for actinobacterial phylotypes from contrasting layers of a lake that were indistinguishable based on 16S rRNA genes [19]. Nevertheless, since our study and these previous works were based on partial sequences, it is highly likely that by using full length 16S rRNA gene sequences the microbia with differing niche requirements can be resolved.

In general, the phytoplankton community composition and the seasonal succession was characteristic for a humic lake with high numbers of small flagellates, including chrysophytes and cryptophytes [49]. For example, as found in our experiments, *Chlamydomonas* sp. is a typical spring bloomer [50], Dinophyceae-taxa can reach a maximum in the spring and autumn [51], and *Gonyostomum semen* is known to form blooms in small forest lakes during autumn [51]. Even though we found phytoplankton to respond in the N+P treatment, the outcome of the experiments was highly dependent on the season and the community composition at the beginning of the experiment.

To conclude, concurrent trajectories in bacterial and phytoplankton communities were observed over the seasonal cycle. The strength of the observed treatment responses was dependent on season and on the level of taxonomic resolution. Differences between the experiments were best explained by seasonal disparities in the bacterial community composition, while within an experiment the differences across treatments were due to differences in the relative abundances of community members. Furthermore, for bacteria there was a clear temporal and functional differentiation inside tribes and thus it seems that, while seasonal variations and treatment responses can be already seen at broad taxonomic levels, ecologically coherent populations are not resolved when using the current definition of freshwater tribes. Our results still highlight the critical importance in ecological studies of obtaining high taxonomic resolution to understand the importance and functioning of complex microbial communities in regards to Liebig's Law of the Minimum.

## Materials and Methods

### Study site

Nutrient manipulation experiments were conducted in Lake Alinen Mustajärvi during the 2009 open water period, at the beginning of May, July and September. The lake is on state land with open access and thus no permits were required for collection of the samples. Further, the location is not protected in any way and the studies did not involve endangered or protected species. Water for the experiments was taken from the upper 1.5 m of the water column where the natural DOC concentration is around 10 mg C L^−1^, but during the period of the experiments it had been elevated to around 12 mg C L^−1^ by monthly additions of cane sugar as part of a parallel project. Each experiment had four treatments (control, +N, +P and +NP) with three replicates for each sampling day. Nutrient additions were made only at the beginning of each experiment with the target rise in concentrations being 0.35 mg L^−1^ for N and 0.05 mg L^−1^ for P. These relatively high additions were necessary to ensure nutrient availability relative to labile DOC throughout the experiments. The nutrient sources used were NH_4_NO_3_ for nitrogen and Na_3_PO_4_ for phosphorus. The water for each experiment was taken with a 30-cm-long acrylic tube sampler (Limnos vol 2 L). Water was sieved with a 50 µm mesh to remove larger zooplankton and mixed thoroughly prior to and after nutrient amendments. 2 L replicates were measured into polypropylene bags, which were then sealed and incubated *in situ* at 0.5 m depth, approximating the effective light climate of the mixed layer of the water column. Each experiment lasted for seven days and sampling was conducted at the start and on days four and seven; here results are mainly reported from day seven.

### Chemical analyses

Analyses of inorganic P and N concentration of the water were made using standard methods (http://www.sfs.fi/). Samples for nutrient analyses were kept on ice and frozen within 4 hours of the nutrient amendments to be analysed later. DOC concentration was analysed from water passed through GF/F filters with a Shimadzu TOC-5000A Total Organic Carbon 140 Analyzer.

### Bacterial abundance and phytoplankton community and biovolume

Bacterial abundance and phytoplankton community composition and biomass were determined from 200 mL samples fixed with 1 mL of Lugol's solution. The phytoplankton were counted by inverted microscopy using a magnification of x400–600; at least 500 counting units (cells, colonies or filaments) in total and at least 50 units of each of the most common taxa were counted. Phytoplankton was identified down to the species if possible; otherwise the genus or a lower taxonomic level was recorded. All individuals were measured and divided into size classes, and the volumes were defined according to the Phytoplankton Register of the Finnish Environment Institute (SYKE). Phytoplankton taxa were divided into autotrophs and potential mixotrophs according to the literature. Some small heterotrophic protists, which were of similar size to phytoplankton, were also counted, excluding ciliates.

Bacterial abundance was determined from samples that were first decolourized with sodium thiosulphate and then stained with DAPI (4,6-Diamino,−2-phenylindole 171 dihydrochloride, Sigma) and filtered onto black polycarbonate filters (Osmonics, pore size 0.22 μm). Ten random fields per filter were photographed with an epifluorescence microscope (Olympus BX60, Olympus 173 Optical Co., Tokyo, Japan) at x1000 magnification and were analyzed with CellC software [52].

### Bacteria community composition

For DNA extraction 100 ml of water from each sample was freeze dried with an Alpha 1–4 LD plus (Christ, Osterode, Germany). The DNA extraction procedure was modified from protocol described by Griffiths et al. [53]. Briefly, freeze-dried material was homogenized with glass beads in a mixture of phenol-chloroform-isoamylalcohol (25∶24∶1) and hexadecyltrimethylammonium bromide. After 5 min incubation on ice to allow humic acids dissolve into PCIAA, tubes were centrifuged. The upper aqueous phase was then re-extracted with chloroform-isoamylalcohol (24∶1), precipitated with polyethylene glycol and dissolved in 50 µL of TE buffer (10 mM Tris [pH 8.0], 1 mM EDTA). Amplification of bacterial 16S rRNA genes (*E. coli* positions 341 to 805) was conducted using general bacteria primers 341F (5′-CCTACGGGNGGCWGCAG-3′) and 805R (5′-GACTACHV GGGTATCTAATCC-3′) [54]. Primer 341F carried a 454FLX adaptor B at the 5′end and primer 805R carried a 5 bp molecular barcode specific for each sample followed by a 454FLX adaptor A at the 5′ end. PCR and amplicon processing prior to sequencing was performed as described in Eiler et al. [55], except for purification of PCR products with Agencourt AMPure XP purification system (Beckman Coulter, Danvers, USA) and amplicon quantification with PicoGreen in a Qubit fluorometer. Equal concentrations of amplicons were sequenced from each sample from adaptor A, using a 454 GS-FLX system (454 Life Sciences, Branford, CT) at the Institute of Biotechnology hosted by the University of Helsinki, Finland. The resulting reads carried the sample-specific molecular barcode and covered the entire V4 region of the 16S rRNA gene as well as flanking regions. The sequencing yielded a total of 97,610 reads. After quality control of barcodes, primer and flowcharts using AmpliconNoise [56], the dataset included 62,330 reads. Of these 1 671 were identified as chimeras using Perseus [56] which left 60,659 reads for further analysis. These sequences were clustered and analyzed based on 97% sequence similarity using UCLUST, but to estimate the impact of resolution level, additional clustering was conducted on 75, 80, 85, 90, 95 and 99% sequence similarities. More details on the analysis are described in [25] including a description of the taxonomic annotation analysis (see also [55]). The 454 sequences have been deposited in the NCBI Short Read Archive under accession number SRA048682.1.

Bacterial community composition was also analysed by length heterogeneity analysis of PCR-amplified 16S rRNA gene (LH-PCR) [23]. LH-PCR was executed and analysed according to [57] with the modifications mentioned in [24]. The phylogenetic affiliations of *Actinobacteria* and *α-* and *β-Proteobacteria* were predicted based on the 16S rRNA gene clone library data from Alinen Mustajärvi (303 clones). For that purpose, a vertical profile of the lake was sampled in summer 2008 and the bacterial community was analysed with LH-PCR and Sanger sequencing. Primers used in LH-PCR were 27f [58] and 518r [59] and in sequencing 27f and 907r [60]. The sequences have been deposited in EMBL database under accession numbers HE616215 – HE616517. From the sequencing results an LH-PCR simulation was conducted according to [57], which gave an interpretation of various LH-PCR marker lengths. LH-PCR fragments with lengths between 466–473 basepairs (bp) were considered as *Alphaproteobacteria*, lengths between 500–508 bp as *Actinobacteria* and lengths between 520–524 bp as *Betaproteobacteria*. Bacterial community composition at phylum level was highly similar when measured with 454 pyrosequencing and the fingerprinting method (LH-PCR). Even though, as stated here, the phylum level does not provide much insight into the metabolic or functional properties of a community, information might be used for community screening, for example for monitoring purposes. LH-PCR was shown to be a fairly reliable predictor of *Actinobacteria* and *Alpha-* and *Betaproteobacteria*. It may also be used for other groups after standardization by sequencing, and as a fast and repeatable method [61] it is well-suited for simple community comparisons.

### Statistical analyses

All statistical analyses were conducted using R ([62]; http://www.R-project.org/). Bacterial α-diversity was estimated with OTU numbers, Pielou's evenness and Chao index [63]–[64] and disparities between seasons and treatments (across experiments due to missing replication) were tested with Kruskal-Wallis rank sum test with post hoc tests. The Morisita-Horn distance measure [65] was used in combination with non-metric multidimensional scaling (NMDS; conducted using function metaMDS in R-package Vegan) to visualize dynamics in community structure (β-diversity) of the data obtained from 454 sequencing, LH-PCR and phytoplankton microscopy, respectively. The sequence data used for NMDS included all OTUs that had more than 20 reads, while LH-PCR data included all the bands that had a sum of area more than 5%. Similarity between NMDS plots for different datasets was tested with procrustes superimposition [66]. Treatment effects on bacterial OTUs and phytoplankton were visualized in heatmaps [67] using standardized number of reads and phytoplankton biovolumes, respectively. Numbers were standardized to maximum number/biovolume of each OTU/taxa. The bacterial heatmap included all OTUs with more than 50 reads and, prior to this analysis, all the samples were randomly re-sampled to the same size based on the sample with smallest sampling size using perl script daisychopper.pl (available at http://www.genomics.ceh.ac.uk/GeneSwytch/Tools.html; [68]). Other analyses were conducted on non-rarefied data. The phytoplankton heatmap included the entire phytoplankton data. Changes in phytoplankton and bacterial community (LH-PCR) following treatments and between seasons (separately and their interactions) were tested with PERMANOVA (permutational multivariate analysis of variance [69], [70]) using function adonis in R.

Bacterial and phytoplankton community dispersion between experiments and treatments were tested with permutational analysis of multivariate dispersions (also called MJ Anderson's permutated analysis of betadispersion), which was applied on Morisita-Horn based dissimilarity matrices (454 data) [28]. Similarity in phylum abundance between 454 pyrosequencing and LH-PCR was also verified using generalized linear models.

## Supporting Information

Figure S1 **General linear model between OTU clustering resolution and pairwise community dissimilarities (Morisita-Horn distances).**
(TIF)

Figure S2 **Proportions of** ***Actinobacteria*** **and** ***Alpha-*** **and** ***Betaproteobacteria*** **in the experiments according to LH-PCR.**
(TIF)

Figure S3 **Biovolume of mixotrophic phytoplankton in the experiments.**
(TIF)

Figure S4 **Chl a, total N, nitrate, ammonium and total P concentrations in the lake during experimental season.**
(TIF)
