# Supplementary figures and images for: Bacterial and Phytoplankton Responses to Nutrient Amendments in a Boreal Lake Differ According to Season and to Taxonomic Resolution
Source: PLoS One. 2012 Jun 8;7(6):e38552. doi: 10.1371/journal.pone.0038552 (PMC3371014; doi:10.1371/journal.pone.0038552)

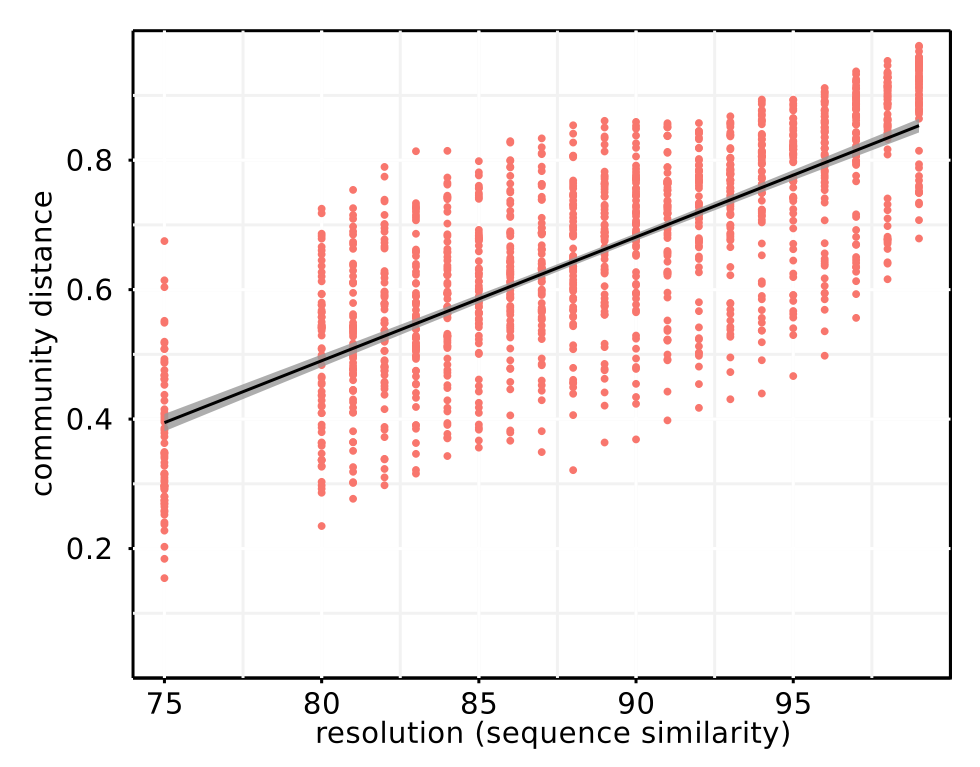

Supplement: Figure S1 — General linear model between OTU clustering resolution and pairwise community dissimilarities (Morisita-Horn distances). (TIF) [file pone.0038552.s001.tif]

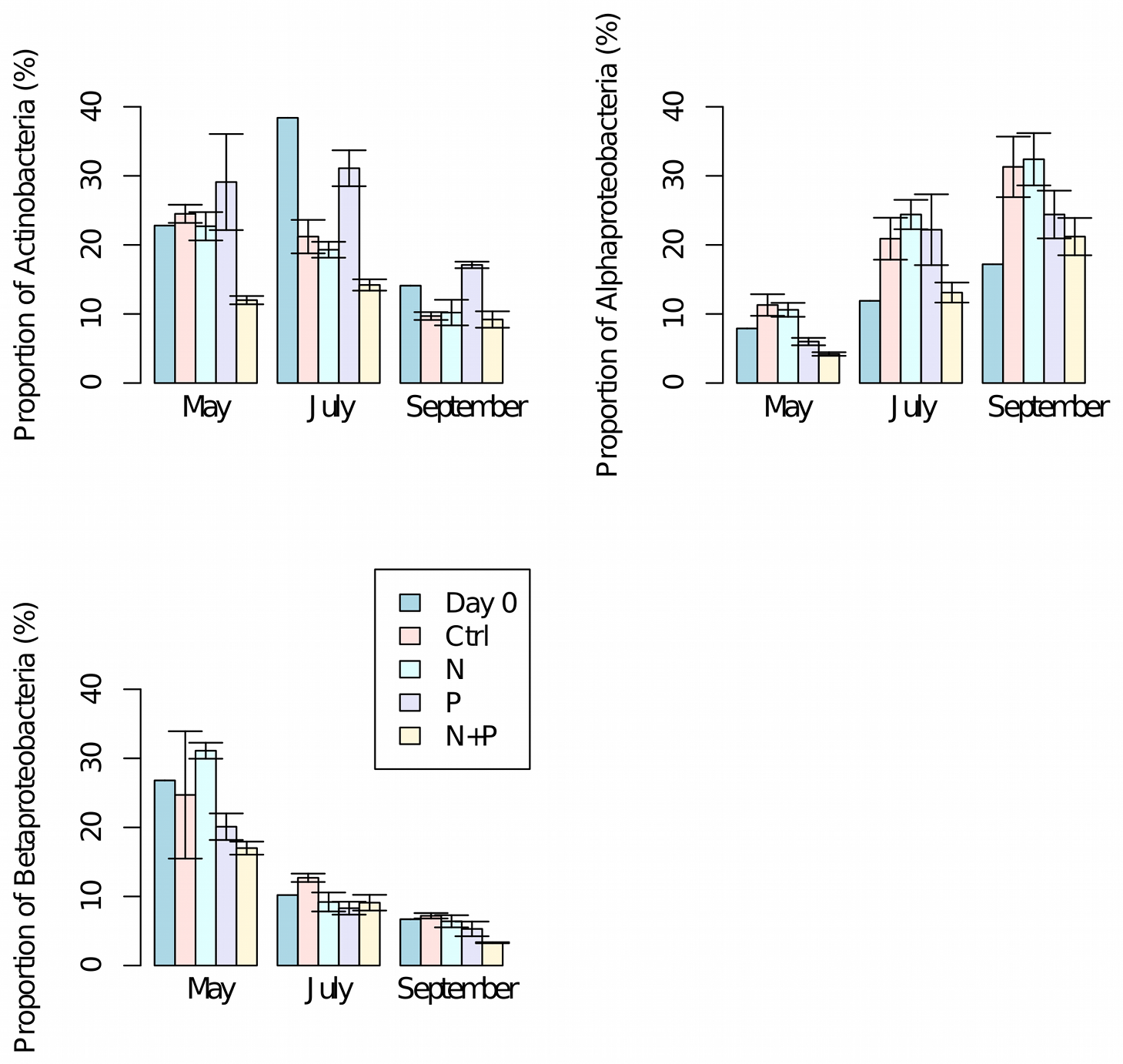

Supplement: Figure S2 — Proportions of Actinobacteria and Alpha- and Betaproteobacteria in the experiments according to LH-PCR. (TIF) [file pone.0038552.s002.tif]

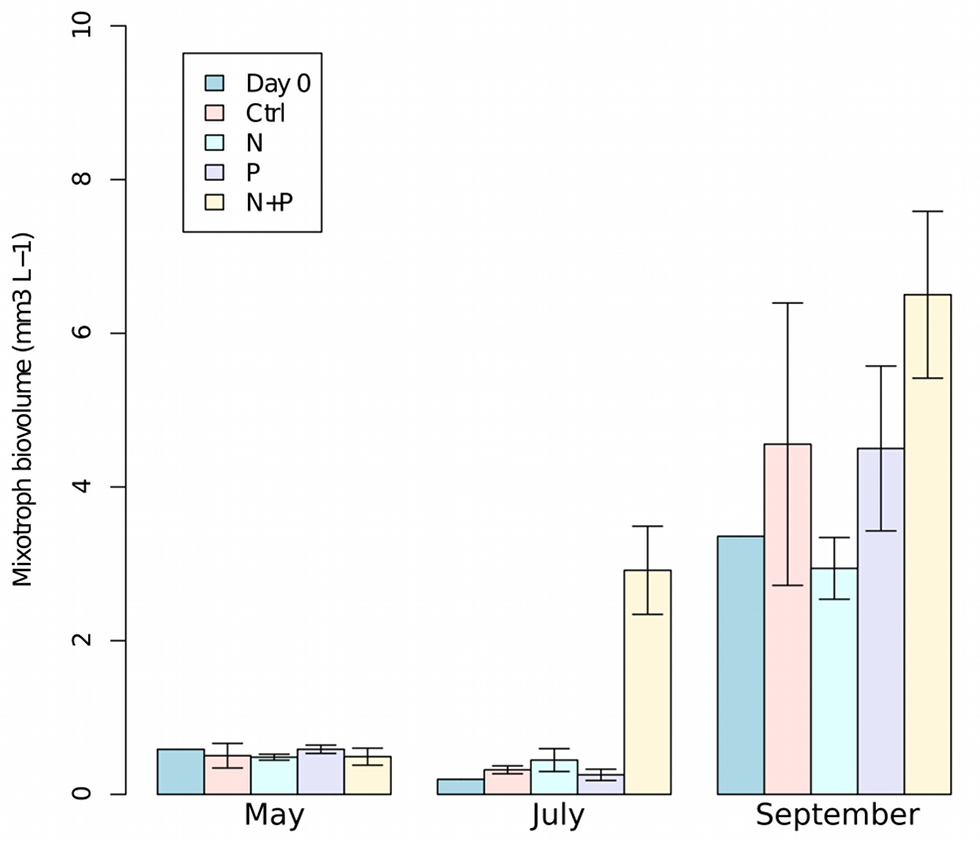

Supplement: Figure S3 — Biovolume of mixotrophic phytoplankton in the experiments. (TIF) [file pone.0038552.s003.tif]

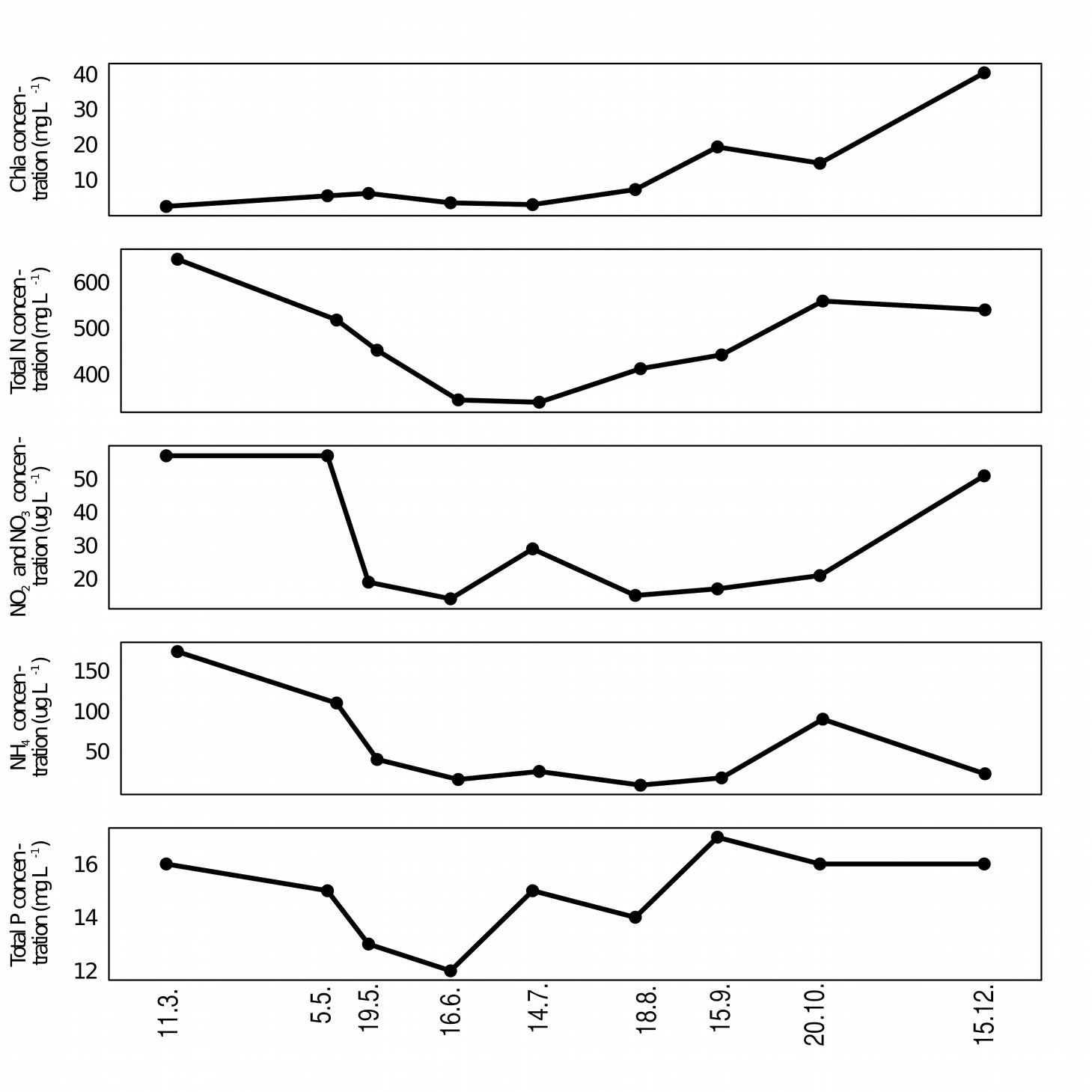

Supplement: Figure S4 — Chl a, total N, nitrate, ammonium and total P concentrations in the lake during experimental season. (TIF) [file pone.0038552.s004.tif]
